# School nurses’ experiences of health-promoting work to prevent stress in Swedish adolescents

**DOI:** 10.3389/fpsyg.2022.933879

**Published:** 2022-07-27

**Authors:** Louise Persson, Charlotta Rahr, Pernilla Garmy, Eva-Lena Einberg

**Affiliations:** ^1^Department of Nursing and Health Sciences, Faculty of Health Sciences, Kristianstad University, Kristianstad, Sweden; ^2^Department of Health Sciences, Faculty of Medicine, Lund University, Lund, Sweden

**Keywords:** adolescents, school nurses, health promotion, stress, experience

## Abstract

**Aim:**

To investigate school nurses’ experiences of health-promoting work to prevent stress in Swedish adolescents.

**Materials and methods:**

Swedish school nurses (*n* = 225) responded to a web survey with open-ended questions. The results were subjected to a qualitative content analysis.

**Results:**

Six categories were identified from the analysis: (1) Knowledge of body and soul, (2) Identifying stress and ill health, (3) Collaborative working within/outside school, (4) Make yourself (i.e., school nurses) visible to the students, (5) Support students’ learning, and (6) Tools for stress management. One of the most important themes in preventing and counteracting stress in school-aged children and adolescents was promoting healthy living habits. That school nurses were easily accessible and visible were important for them to identify stress as early as possible. The close collaboration with the school health care team and building relationships with the students were emphasized. The stress in adolescents was largely linked to schoolwork and performance anxiety. To counteract this, the dialogue with the teachers was important to increase their knowledge of mental health problems and stress and influence the scheduling of school exams.

**Conclusion:**

The study contributes to increased knowledge in the field and provides concrete strategies for how school nurses can work to counteract stress in students.

## Introduction

Mental health problems among adolescents are increasing (Bor et al., 2014), and the prevalence of recurring psychosomatic symptoms has doubled since the 1980s (Högberg et al., 2020). Without recovery and rest, the risk of depression and fatigue syndrome increases with prolonged stress (Åsberg et al., 2010). According to Selye (1965), stress is a reaction to a stimulus that can have a positive or negative effect, depending on the context. Stress is present throughout the period that an individual is exposed to various unspecified stressors. Furthermore, there is also a difference between acute and persistent stress (Tan and Yip, 2018). When people are exposed to stress, they go through several stages. The first stage is the alarm stage, where the body is put on standby and prepares for any danger; the second stage is the resistance stage, where the individual tries to achieve balance by avoiding the change and adapting to the situation. The last stage is the stage of exhaustion, which occurs when the resistance to counteract the stressors becomes too great (Tan and Yip, 2018). Stress can have positive and negative effects, where positive stress is necessary for certain situations for the individual to survive. On the other hand, negative stress can affect several of the body’s organs. The brain can be affected when exposed to long-term stress, for example, problems with memory and learning. The heart is also affected by acute and prolonged stress, leading to increased heart rate and sympathetic nervous system activation. Stress also affects the immune system, appetite, gastrointestinal functions, and endocrine system (Yaribeygi et al., 2017). Everyone can be exposed to stress and experience depression or severe fatigue. The balance between what is considered common and disease is difficult to discern. There are no obvious limits. Stress is linked to mental illness in several different ways. If it is long-lasting and the possibility of rest is limited, it can give rise to symptoms of irritation, fatigue, depression, and anxiety (Åsberg et al., 2010).

### Stress in adolescents

Adolescents show different symptoms and signs of stress depending on age. Common symptoms are difficulty concentrating, changes in sleep and eating habits, and changes in mood and behavior. Headaches, abdominal pain, and general pain are also common symptoms. Other symptoms include difficulty falling asleep, concentrating, depression, procrastination, and melancholy (Warghoff et al., 2020: Hörbo et al., 2021; McKegney, 2021). McKegney (2021) reports that isolation, increased anxiety, and avoidance of leisure activities are warning signs. According to Jakobsson et al. (2019), stress is the most common cause of adolescent sleep problems. The concept of stress includes school stress, everyday stress, and fear of missing out. School stress can manifest as anxiety, constant agitation, stress over schoolwork, and not keeping up (Jakobsson et al., 2019). According to Kyoung (2019), adolescents who perform worse in school are also those who, to a greater extent, show signs of depression or suicidal thoughts.

Other stressors may be conflicts in close relationships and new environments, such as changing schools or classes (Kyoung, 2019; Warghoff et al., 2020). Jakobsson et al. (2019) and Warghoff et al. (2020) report that young people set demands and expectations on themselves that they cannot always live up to. Parents and teachers also have some expectations of adolescents that can contribute to stress. According to Mörelius (2015), the school nurse should be aware of when adolescents seek contact for “simple” problems as this may be a sign that they want contact with an adult who can listen and provide support.

### The role of the school nurse

School nurses are employed at every school in Sweden, however, schools with few students (approximately less than 300–400 students) might not have access to the school nurse every day of the week. In average, each school nurse is responsible for 484 students (Ellertsson et al., 2017). According to Swedish law (Education Act, 2010), school health care must primarily focus on prevention and health promotion. Students have the right to medical, psychosocial, psychological, and special educational interventions. The National Board of Health and Welfare (2017) states that good collaboration between school health care and teachers at the school creates good conditions for the work. School health care aims to create as good conditions as possible for a good learning situation. According to the Education Act (2010), students must be offered at least three health talks in compulsory school and one in upper secondary school. Hilli and Pedersen (2021) report that these health conversations are a tool where school nurses can identify ill-health and young people’s specific needs by implementing appropriate measures. The school nurses have an important task in supporting and giving young people the opportunity to trust their abilities. To improve health and well-being, it is important to listen to adolescents’ expressed feelings and needs, both the expressed and the unspoken (Hilli and Pedersen, 2021). School nurses must be visible to give their students good support and know who they can turn to. Accessibility can be challenging; therefore, school nurses must reflect on their role to be “enough” (Sherwin, 2016). School nurses must be flexible and open to implementing measures early and collaborate with the school health care team and guardians (Hilli and Pedersen, 2021). Many school nurses consider their work with adolescents with mental health problems meaningful and important (Jönsson et al., 2019). School nurses experienced a transition to a more digital way of working during the COVID-19 pandemic (Martinsson et al., 2021). The current study aimed to investigate school nurses’ health-promoting work to prevent stress in Swedish adolescents.

## Materials and methods

The current study was conducted among school nurses in Sweden in 2021 during the coronavirus 2019 (COVID-19) pandemic. In Sweden, schools for 6–15-year-old children were mainly open during the pandemic. However, schooling for 16–19-year-old adolescents was partly by distance learning. The study was conducted as an anonymous cross-sectional study with a qualitative approach in the form of an online survey with open-ended questions. Before data collection started, the study was approved by the Swedish Ethical Review Authority (2021-00946).

### Sample

Purposeful sampling was applied (Polit and Beck, 2017). The online survey was distributed to school nurses in Sweden who worked in school health care, regardless of whether the school was private or public. The inclusion criteria for participating in the study were to work as a school nurse in Sweden. The school nurses who participated worked in primary and secondary schools with varying ages for the students, between 6 and 19 years old.

### Procedure and data collection

Initially, a short request to school nurses to participate in the study through a post in the Facebook group “School nurses in Sweden,” with 2,600 members. The second approach meant that an information letter was sent to the school health care administration of schools in southern Sweden, who then forwarded the information letter with the link to the web-based survey to the school nurses in their schools. A reminder was sent out on one occasion after 2 months. The survey consisted of four open-ended questions aimed at the school nurse’s health-promoting work, see Appendix. The questionnaire was available to answer from May to December 2021.

### Analysis

The open-ended questions were analyzed with inductive, qualitative content analysis. According to Graneheim and Lundman (2004), qualitative content analysis focuses on distinguishing variations in the data material. Furthermore, they describe that similarities and differences can be formulated in categories and themes. The current study analyzed the texts on the open-ended questions by establishing an analysis schedule. The analysis scheme created columns with sentence units, codes, subcategories, and categories (Table 1). The questionnaire responses were placed in numerical order in the sentence units column. The meaning units were read several times, and codes were worked out. These can be likened to labels, where one or more words summarize the text. Graneheim and Lundman (2004) describe the codes as tools to help the authors reflect on the text. Once the codes were entered into the schedule, mind maps were used to distinguish subcategories and categories for the different answers. Graneheim and Lundman (2004) describe that the categories consist of several codes with similar meanings. After further analysis and sorting of similarities and differences, these were further changed before subcategories emerged that became a latent interpretation of the content of the text. The analysis was discussed between the four authors until a consensus was obtained.

**TABLE 1:** Overview of the sentence units, codes, subcategories, and categories.

Sentence units	Codes	Subcategories	Categories
Shares knowledge about stress, how it works, how it affects the body and what can affect stress	Informs about stress and how it can affect the body	Sharing knowledge about stress and body	Knowledge of body and soul
Trying to create relations with the students to enable them to visit me for various reasons, not only bodily complaints.	Creating relations enables the students to visit the school nurse when needed.	Building relationships	Make yourself (i.e., school nurses) visible to the students
Collaboration between the school health care and the teaching teams about how to discuss stress and mental health in the classroom.	Working together with the teachers, school administration and the school health professionals	Collaboration with the school health team and teachers	Collaborative working within/outside school

## Results

Six categories were identified from the analysis: (1) Knowledge of body and soul, (2) Identifying stress and ill health, (3) Collaborative working within/outside school, (4) Make yourself (i.e., school nurses) visible to the students, (5) Support students’ learning, and (6) Tools for stress management.

### Knowledge of body and soul

The school nurses expressed that an important part of their work was preventing stress and talking to the students about healthy living habits. The students’ screen time and its connection to poor sleep were highlighted in the health conversations and the importance of a regular circadian rhythm. Conversations about physical activity and good eating habits promote students’ stress tolerance. For students to balance school and leisure, it was considered important that they were allowed to rest and recover, even though it was also important to do fun things in their free time. The school nurses gave the students advice on how they could affect their health independently. While some school nurses mainly talked about healthy habits during the individual health conversations, others went out to the classes alone or with the school social worker. The lectures in class could, for example, be about stressful lifestyles and alcohol, drugs, and sex education. Topics discussed depended largely on the students’ age, and the information was adapted to the students’ maturity. To make it easier to catch the students’ attention, some of the school nurses divided the class into small groups instead of informing everyone at the same time. To give students an increased understanding of what happens in the body during stress, the school nurses informed them about the difference between positive and negative stress and its effect on the body.

*“I talk a lot about sleep, activity and diet with the adolescents to counteract the negative stress at school. With the right conditions, you can deal with stress better, in my opinion.”*

The school nurses also expressed that students’ mental health problems have increased in recent years. When they talked about mental health, the school nurses also tried to convey that the mood varies over time, that all bad mood is not anxiety and depression, and that everyone can have bad days without a diagnosis. Many school nurses consider it important to talk about the students’ thoughts about the future, prepare them for adulthood, and strengthen their self-esteem. Some school nurses tried to have a salutogenic perspective and encourage health factors when discussing mental illness.

*“The whole school health care team (not just the school nurse) should focus on normalizing that the mood varies over time. All bad mood is not anxiety and depression.”*

### Identifying stress and ill-health

The health conversation and spontaneous visits were good opportunities to capture abnormalities and identify mental and physical health problems. During these conversations, the school nurse tried to work with the underlying cause of the student, for example, having a headache and what stressors there were. During the health visit, the school nurse detected early signs of stress and mental illness and implemented the necessary measures. They also followed up on any deviations and thus worked preventively to counteract ill health. Length, weight, vision, and hearing were checked during the health visit, and vaccinations according to the basic program were also performed.

*“Trying to capture them in the health conversation and talk about stress, what stress is, and that stress is part of life.”*

School attendance could be improved by detecting health problems early, such as relationship problems with friends. Capturing those students who already had high school absenteeism was also a way of identifying mental illness.

*“Capturing students who are not feeling well mentally at an early age.”*

### Collaborative working within/outside school

Collaborating with other professionals was one of the most important tasks to prevent ill health and stress. It was important for the school nurses to collaborate with the school health care team and the teachers at the school. The stress in adolescents was largely linked to schoolwork and performance anxiety. To counteract this, the dialogue with the teachers was important to increase their knowledge of mental health problems and stress and influence the scheduling of the number of tests per week. The school nurses often mentioned the school social worker as the closest partner in the school health care team. At the school health care meetings, the school nurse also had the chance to highlight important information from the health surveys and share it with the team members. In this way, they could together map students who needed, for example, a special education need coordinator or school social worker. When there was a risk of stress and ill health, the relationship with the child’s parents was greatly important to the entire school health care team. The school health care team was present at many school parent meetings. This was considered a good opportunity to explain all professional functions’ tasks.

To provide the right care and support to those students with illness, harmful stress, or disability, collaboration with other health services agencies (such as child and adolescent psychiatric services) was important. It also emerged that collaboration with the student council was an excellent opportunity to hear the students’ voices.

*“Collaboration between school health care team and the teachers on how the group can talk about stress and mental health in the classrooms.”*

*“Follow up if needed, call home to parents, or refer to the school social worker, child and adolescent psychiatric service or other instance if relevant.”*

### Make yourself (i.e., school nurses) visible to the students

Accessibility and always having the door open were an important part of the school nurses’ work in health promotion. Students always felt welcome when the need arose, and the “drop-in” visits helped build relationships. Being visible at school, for example, being outside in the school yard during breaks and inside in the school canteen during lunchtime to identify problems and to establish a connectedness with the students, was also an important task. Trusting relationships with the students was valuable because detecting deviations and identifying mental and physical illnesses were easier. Trust made the students dare to open up and talk about difficulties. The duty of confidentiality was also mentioned that strengthen the relationship and contribute to security. The school nurse and others in the school health care team confirmed the students, put them in the center and captured them by listening and giving support.

*“Always keep the door open so they can come and talk.”*

*“Listening in and being there as support. Be able to advise on health without judging. Health visits and be available for spontaneous visits.”*

### Support students’ learning

According to the school nurses, it was important to influence the students’ work environment to give them the right conditions to complete their studies, as many students felt stressed and had performance anxiety over schoolwork. Many stated that there were shortcomings in the scheduling at school and that it was not unusual to have two or three tests the same week. This meant a lot of stress, and the close collaboration with the teachers was important to develop a good study plan for exams and assignments. The school nurse expressed an increased understanding of mental illness and how it could affect school performance in school health care. Therefore, close collaboration was extremely important for the students to assimilate their studies well and achieve a reasonable workload and a good work environment. It also became easier to identify the students who needed to be supported with resources to achieve their goals in school.

*“Trying to influence via the school health care team at school management level so that teachers can assess the students’ abilities in other ways than through tests and that teachers communicate better in their work teams when planning their subjects. It is not uncommon for students to sometimes have 2 tests on the same day or 3 tests in the same week.”*

### Tools for stress management

The school nurse gave the students tools to identify stress and how stress can affect them. Some schools worked with coaching and structure in everyday life. In other schools, the school nurses and school social workers had relaxation exercises in groups in the form of yoga, mindfulness, or other breathing exercises, which was much appreciated. The school nurses could also help the student by sketching out a mind map to visualize what a day looked like.

*“I work with mindfulness, yoga, and relaxation in small groups (when it’s not corona).”*

*“Counteract stress with painting and other activities during class time. Get to know how I study best. Study - pause- study -pause. Get on the so-called “the sleep train,” stop using the phone before bedtime, use of weight blanket, foot massage. breathe, have a positive mindset, to be good as you are.”*

## Discussion

The study revealed several aspects of the Swedish school nurse’s health promotion work to prevent stress in adolescents. Three main findings will be highlighted. First, the school nurses describe healthy living habits as a prerequisite for preventing stress. Second, it is important for the school nurse to be visible and accessible to create good relationships with the students. The third finding addresses collaboration with other actors in and outside the school.

Healthy living habits are a prerequisite to counteracting stress. A key finding is the school nurses’ conversations about healthy living habits to prevent adolescent stress. Schultchen et al. (2019) described that those who were more physically active often had a lower level of stress and vice versa, that higher stress levels were often linked to low physical activity. Furthermore, Schultchen et al. (2019) found that a similar connection could not be made between a healthy diet and stress, as what was considered a healthy diet was individual and, in many cases, emotionally driven. Some ate when they were stressed, while others could not eat when they experienced stress. In the current study, the school nurses believe that reduced screen time is a prerequisite for good sleep as poor sleep habits increase sensitivity to stress. Åsberg et al. (2010) confirmed this and argued that rest and recovery were important to avoid the negative effects of stress. In their study, Hale and Guan (2015) described that social media affected adolescents’ sleep, partly because they fall asleep later but also decreased their sleep quality. Woo et al. (2021) were of a different opinion and emphasized in their study that screen use had positive effects, for example, on mental health. Internet functions could strengthen social support and the feeling of belonging, which, according to Antonovsky (1987), is important for preventing and managing stress. However, one consequence of using social media may be that physical encounters are avoided, leading to reduced physical activity and reduced stress tolerance.

The school nurses emphasize the importance of being accessible and visible to create good student relationships. Our study shows that school nurses believe that the possibility of students accessing the school nurse on “drop-in” visits allows them to identify stress and ill health in adolescents. Hilli and Pedersen (2021) described that it was important for the students to feel confident in the school nurse and feel they could talk about everything. It was also important as a school nurse to listen to what the students said and interpret what was not said. Sherwin (2016) confirmed this, which helped the students know who to turn to, but also emphasized that it was important that the school nurse during the “drop-in” visits reflected on her role as accessibility could sometimes be a challenge (Sherwin, 2016). The school nurse is obliged to follow the national program with regular health conversations and health check-ups and vaccinations. However, being visible and accessible to the students is also important. To balance these two important areas, which identify stress and ill health in adolescents, the school nurse must be given the time and space needed.

Collaboration between different actors is important for the school nurse in promoting work against stress. Typical collaborations within schools are with the school social worker and the teachers, and outside schools with the adolescent health care facilities and the child psychiatric clinic (Jönsson et al., 2019; Martinsson et al., 2021). Hilli and Pedersen (2021) described the importance of a good collaboration with the school health care team and guardians to implement measures in the event of ill health at an early stage. The collaboration with the guardians and their commitment was also important to the health workers to promote the students’ health. Furthermore, they considered that the school nurse and the teachers complimented each other and that the school nurse’s competence was required when teaching specific health subjects, which the students appreciated (Hilli and Pedersen, 2021).

School nurses believe that students often experience stress and performance anxiety related to schoolwork. Many of the school nurses express in the study that it is not unusual to have several tests in the same week, which increases student stress. The results also show that it is important to coordinate the study plan with the teachers, which gives the teachers an increased understanding of the students’ situation. Jakobsson et al. (2019) confirmed this in their study and reported that many students experienced stress in connection with school work and that it took a lot of time to prepare for tests and school assignments. Students sometimes had to sit late into the night, which affected sleep (Jakobsson et al., 2019). Against this background, a coordinated study plan must be highlighted at the school management level to reduce students’ stress and performance anxiety and get all actors to work toward the same goal.

### Strengths and limitations

The study’s strengths are the relatively high number of participants and the wide variety of data. But there are approximately 3,000 school nurses in Sweden, and less than 10% of this population has responded to the survey. Due to the anonymous and voluntary data collection, we do not know if the included respondents are representative of the whole school nurse population in Sweden. School nurses’ work situation differs worldwide, so the generalization of the results may be limited. However, other professionals also work with health promotion to prevent stress in adolescents, and the experiences of Swedish school nurses could provide guidance and inspire other settings.

### Implications for practice and suggestions for future research

The Swedish school nurses and health care team actively counteract and prevent stress. The results also showed that collaboration was important to the school nurse’s work, both within school with other school staff, and also outside school with other health agencies. The study also shows that it is important for school nurses to be visible and accessible to identify ill health and stress more easily. One suggestion may be for the school nurse to set aside days in their calendar that the students are aware of, specifically dedicated to “drop-in” visits. Suggestions for future research are evaluations of school nurses’ health promotive work to prevent stress in adolescents. It would also be valuable to investigate school nurses’ experiences of health promoting work in other countries and regions.

## Conclusion

According to the Swedish school nurses, healthy living habits were central to preventing and counteracting stress. School nurses felt that being easily accessible and visible were important to identify stress as early as possible. The close collaboration with the school health care team and building relationships with the students were emphasized. The stress in adolescents was largely linked to schoolwork and performance anxiety. To counteract this, the dialogue with the teachers was important to increase their knowledge of mental health problems and stress and influence the scheduling of the number of tests per week. The study contributes to increased knowledge in the field and provides concrete strategies for how school nurses can work to counteract stress in students.

## Data availability statement

The raw data supporting the conclusions of this article will be made available by the authors, without undue reservation.

## Ethics statement

The studies involving human participants were reviewed and approved by Swedish Ethical Review Authority (2021-00946). The patients/participants provided their written informed consent to participate in this study.

## Author contributions

PG and E-LE: conceptualization, funding acquisition, project administration, resources, and supervision. LP and CR: writing–original draft. All authors: data curation, formal analysis, investigation, methodology, writing–review and editing, and approved the submitted version.

## Appendix

1. What main task do you think you have to promote the health of school children in your role as a school nurse?
2. What health promotion efforts should school health care focus on?
3. At what age do you think stress is most common?
4. How do you preventively work as a school nurse to counteract stress?
